# Identification and Validation of a Novel Six-Gene Expression Signature for Predicting Hepatocellular Carcinoma Prognosis

**DOI:** 10.3389/fimmu.2021.723271

**Published:** 2021-12-01

**Authors:** Zongcai Yan, Meiling He, Lifeng He, Liuxia Wei, Yumei Zhang

**Affiliations:** ^1^ Department of Medical Oncology, Guangxi Medical University Cancer Hospital, Nanning, China; ^2^ Department of Oncology, Ruikang Hospital Affiliated to Guangxi University of Chinese Medicine, Nanning, China

**Keywords:** hepatocellular carcinoma, prognosis, risk score, tumor microenvironment, biomarkers, differential gene expression analysis

## Abstract

**Background:**

Hepatocellular carcinoma (HCC) is a highly lethal disease. Effective prognostic tools to guide clinical decision-making for HCC patients are lacking.

**Objective:**

We aimed to establish a robust prognostic model based on differentially expressed genes (DEGs) in HCC.

**Methods:**

Using datasets from The Cancer Genome Atlas (TCGA), the Gene Expression Omnibus (GEO), and the International Genome Consortium (ICGC), DEGs between HCC tissues and adjacent normal tissues were identified. Using TCGA dataset as the training cohort, we applied the least absolute shrinkage and selection operator (LASSO) algorithm and multivariate Cox regression analyses to identify a multi-gene expression signature. Proportional hazard assumptions and multicollinearity among covariates were evaluated while building the model. The ICGC cohort was used for validation. The Pearson test was used to evaluate the correlation between tumor mutational burden and risk score. Through single-sample gene set enrichment analysis, we investigated the role of signature genes in the HCC microenvironment.

**Results:**

A total of 274 DEGs were identified, and a six-DEG prognostic model was developed. Patients were stratified into low- or high-risk groups based on risk scoring by the model. Kaplan–Meier analysis revealed significant differences in overall survival and progression-free interval. Through univariate and multivariate Cox analyses, the model proved to be an independent prognostic factor compared to other clinic-pathological parameters. Time-dependent receiver operating characteristic curve analysis revealed satisfactory prediction of overall survival, but not progression-free interval. Functional enrichment analysis showed that cancer-related pathways were enriched, while immune infiltration analyses differed between the two risk groups. The risk score did not correlate with levels of PD-1, PD-L1, CTLA4, or tumor mutational burden.

**Conclusions:**

We propose a six-gene expression signature that could help to determine HCC patient prognosis. These genes may serve as biomarkers in HCC and support personalized disease management.

## Introduction

Liver cancer is one of the most common types of malignancies and is associated with a high mortality rate. In 2015, liver cancer ranked sixth in the global incidence of cancer and fourth among the most deadly tumors (1). Chronic hepatitis B virus infection is the leading cause of hepatocellular carcinoma (HCC) in Asia, while chronic hepatitis C virus, alcoholic cirrhosis, and non-alcoholic steatohepatitis are the primary causes in Western countries (2). Since symptoms of HCC often present when the disease has reached an advanced stage and therapeutic strategies are limited, the five-year survival rate of HCC patients remains low. Standard therapies for treating advanced HCC are systemic chemotherapy and molecular targeted therapies (2, 3). The poor prognosis of HCC is due mainly to metastasis, poor liver function, and deteriorating overall physical condition (4–6). Therefore, there is an urgent need to develop new prognostic markers for HCC to predict patient outcomes.

The main cellular components in the HCC microenvironment consist of immune cells, fibroblasts, macrophages, and cancer stem cells (7). The tumor microenvironment plays a crucial role in tumor cell survival, growth, proliferation, epithelial–mesenchymal transition, and metastasis (8). Understanding the role of this microenvironment in metastasis is important for the development of anticancer therapies. For example, immune checkpoint inhibitors (ICIs) have revolutionized the field of tumor therapy. The most widely studied checkpoints are the programmed death protein 1 (PD-1), programmed death receptor ligand 1 (PD-L1), and cytotoxic T-lymphocyte antigen 4 (CTLA-4), all of which play a role by blocking the interaction between the inhibitory receptors expressed on T cells and their ligands (9). At present, some ICIs have been approved by the United States Food and Drug Administration for clinical use. However, relatively few HCC patients benefit from such inhibitors (10). Thus, an effective scoring system is needed for evaluating the proportion and role of different immune cell subtypes in the tumor microenvironment.

The pathogenesis of HCC is complex. Disease diagnosis and management can be guided by clinical diagnostic indicators, including biomarkers such as alpha-fetoprotein, features on computed tomography or magnetic resonance images, pathological biopsy, and staging index (based on tumor size, vascular or lymph node invasion, and distant metastasis). However, due to the great heterogeneity of HCC, the same treatment may lead to different outcomes in different patients. Next-generation sequencing can help elucidate and clarify complex molecular mechanisms in cancers such as HCC, aiding the design of more effective therapies.

Therefore, in the present work we used bioinformatic techniques to perform comprehensive gene expression analysis and identify potential prognostic markers of HCC. We used publicly available gene expression datasets from The Cancer Genome Atlas (TCGA) and the Gene Expression Omnibus (GEO) databases to establish a six-gene prognostic signature (PZP, HMMR, LCAT, GRAMD1C, LPL, and ANGPTL1). We validated the signature against an independent dataset from the International Genome Consortium (ICGC). We further explored correlations among the signature-based risk score, tumor mutational burden and levels of PD-1, PD-L1, and CTLA4. Importantly, gene set enrichment analysis (GSEA) was used to understand the functional annotation of signature genes, and the role of those genes in the tumor microenvironment was investigated by analyzing tumor-infiltrating immune cells.

## Materials and Methods

### Data Collection From TCGA, GEO, and ICGC Databases

Liver hepatocellular carcinoma (LIHC) RNA-sequencing data (in terms of raw read counts and FPKM) were downloaded from TCGA database (https://gdc.cancer.gov/). The corresponding clinical and survival data were downloaded from the UCSC Xena browser (https://xenabrowser.net/) under the cohort name “TCGA Liver Cancer (LIHC)” (19 datasets). Somatic mutation data of TCGA-LIHC were obtained from TCGA. We selected the data of “masked somatic mutation” processed by the VarScan software from the four subtypes of the data files on TCGA website (11). The primary HCC tumor tissues and adjacent non-tumor tissues of datasets GSE54236 and GSE104310 were obtained from the GEO database (https://www.ncbi.nlm.nih.gov/geo/), a free database of microarray profiles and next-generation sequencing. The microarray data of GSE54236 included 81 tumor tissues and 80 adjacent non-tumor tissues (12). The RNA-sequencing data of GSE104310 (FPKM value) were generated on the GPL16791 platform (Illumina HiSeq 2500), including 12 tumor tissues and eight adjacent non-tumor tissues. The NCBI-GEO database only provides FPKM values for each sample in GSE104310. The LIRI-JP RNA-sequencing data and its corresponding clinical information were downloaded from the ICGC portal (https://icgc.org/) (13). In addition, the survival data for the ICGC cohort contains only overall survival information. Since data were downloaded from publicly available databases, our institution waived the requirement for ethical approval.

### Processing of RNA-Sequencing and Microarray Data

In TCGA dataset, we retained the tissue type of the primary tumor (only unique samples of type “TCGA-##-##-01A” indicating primary tumor) and normal control tissues (only samples with a “TCGA-##-####-11A” ID), finally obtaining 369 tumor tissues and 50 normal control tissues. In the ICGC dataset, we deleted genes for which 20% of values were missing, and the remaining missing values were imputed using the weighted K-nearest neighbor algorithm with K = 10 in the “DMwR” package (14). The FPKM values from TCGA, ICGC, and GSE104310 were first transformed into transcripts per million (TPM) and transformed by log_2_(x + 1). The missing values in the GSE54236 dataset were filled using the “impute” package in R and the KNN algorithm with K = 10 (15). After collection of TCGA clinical data from the UCSC Xena browser, 339 patients with complete information on survival time, survival status, and tumor node metastasis (TNM) stage were selected for the survival analysis. In the end, 339 patients from TCGA cohort were assigned to the training cohort, while 231 patients from the ICGC cohort were included as the external validation cohort.

### Analysis of Differentially Expressed Genes (DEGs)

We used the “biomaRt” package (version 2.44.4) *via* the Ensembl database to transfer Ensemble IDs to gene symbols in TCGA-LIHC data (16, 17). We defined the maximum expression value as the expression level of the gene symbol when several Ensemble IDs corresponded to one gene symbol. After removing duplicate gene symbols, we annotated 55,349 genes. Differential gene expression analysis in the TCGA-LIHC dataset was performed using the “edgeR” package (18, 19), while analysis of GSE104310 and GSE54236 was conducted using the “limma” package (20). The raw read count matrix of TCGA-LIHC was used to identify DEGs between 369 tumor tissues and 50 adjacent non-tumor tissues with the “edgeR: package. To be included in DEG analysis, we defined that the count per million for gene expression had to be higher than 0.5 in at least 50 samples. In this way, 18,512 genes were selected for subsequent DEG analysis. The “limma” package was used to identify DEGs in GSE54236 and GSE104310. DEGs with an absolute log_2_(fold change) > 1 and a Benjamini–Hochberg adjusted *P-*value < 0.05 (21) were considered in further analyses. The overlapping DEGs among the three datasets were considered the final DEGs.

### Identification and Validation of a Prognostic Gene Expression Signature

DEGs common to the three datasets were identified using a Venn diagram. The potential prognostic genes were screened by Kaplan–Meier analysis and univariate Cox regression based on overall survival and a definition of significance of *P <* 0.05. LASSO Cox regression was conducted in the training cohort using the “glmnet” package based on the intersecting genes common to the Kaplan–Meier and univariate Cox analyses (22, 23). The best penalty parameter lambda (λ) was confirmed by 10-fold cross-validation (24). Based on the optimal λ, we obtained a list of potential prognostic genes. The potential prognostic genes identified by LASSO regression were entered into the multivariate Cox both-direction stepwise regression model. A multivariate Cox model was established according to the lowest Akaike Information Criterion value by using the *step* function in the “stats” package in R (25). Assumptions for the multivariate proportional hazards modeling were checked using the “survival” package in R (26). The multicollinearity of covariates was estimated through the variance inflation factor (VIF), and we defined that VIF ≥ 2 was considered to indicate multicollinearity in the study. Genes satisfying the assumption of proportional hazard (*P >* 0.05) and VIF < 2 were selected to reconstruct the multivariate Cox regression model. Finally, a multivariate Cox regression model with regression coefficients was obtained based on gene expression and patient survival data. All the covariates in the multivariate Cox regression model satisfied proportional hazards assumptions and the global Schoenfeld test (*P* = 0.4185).

The risk scores of each patient were calculated according to the formula: $risk\;score=\Sigma_{i=1}^{n}(coef_{i}*Expression_{i})$ . The **“**coef**”** derived from the multivariate Cox regression was the regression coefficient of the gene, and **“**Expression**”** indicated the gene expression in terms of log_2_(TPM + 1).

Patients in TCGA cohort were divided into high- or low-risk groups based on the optimal cut-off value (1.195033) of the risk score derived from the *surv_cutpoint* function in the “survminer” package in R (27). This cut-off value was also used as the threshold to stratify patients into high- or low-risk groups in the ICGC validation cohort. Kaplan–Meier analysis, Cox, decision curve analysis, and time-dependent receiver operating characteristic (ROC) curve analyses were conducted to evaluate the prognostic value of the gene expression signature in TCGA cohort. These same operations were repeated in the ICGC cohort to assess the robustness of the multivariate Cox model.

Principal component analysis (PCA) and t-distributed stochastic neighbor embedding (t-SNE) were used to explore the distribution of the two different groups based on the expression [log_2_(TPM + 1)] of the six genes of the signature (25, 28). Default parameters were used, except for perplexity = 10 and max iter = 500. The same parameters were used for all datasets. The “finalfit” package was used to perform univariate and multivariate Cox analyses (29). The predictive power of the gene expression signature was evaluated by time-dependent ROC curve analysis using the “survivalROC” package (30).

### Tumor Mutational Burden

Tumor mutational burden, a newly established independent predictor of the efficacy of immune checkpoint inhibitors, is defined as the number of mutations per megabase of genomic territory (31, 32). Tumor cells with higher tumor mutational burden are more susceptible to immune responses, so such patients are more likely to benefit from immunotherapy. We used the *read.maf* function to read the MAF file, and the tumor mutational burden level of each patient in the MAF file was calculated using the *tmb* function in the “maftools” package. The correlation between tumor mutational burden and risk score was analyzed by the Pearson correlation test, and we explored the correlations between risk score and the expression [log_2_(TPM + 1)] of three immune checkpoint proteins (PD-1, PD-L1, and CTLA-4). In addition, we explored the expression of the six-gene expression signature using TCGA cohort raw read count format, and differential expression was assessed using the “edgeR” package.

### Gene Set Enrichment Analysis

To investigate the functions and pathways of DEGs between the high- and low-risk groups in TCGA cohort, GSEA was performed using GSEA software (v4.0.3) (https://www.gsea-msigdb.org/). The TPM matrix of TCGA cohort consisted of 55,317 genes and 339 samples for GSEA analysis. The Hallmark (v7.2) and KEGG (v7.2) gene sets were collected from the Molecular Signatures Databasev7.2 page (https://www.gsea-msigdb.org/gsea/downloads.jsp) as functional gene sets. Gene sets with normalized enrichment score > 1 or < 1, nominal *P* value (*P*) < 0.05, and false discovery rate (*q*) < 0.05 were considered statistically significant.

### Correlation of the Risk Score With the Proportion of 28 Types of Tumor-Infiltrating Immune Cells

We conducted ssGSEA to evaluate the correlation between the risk score and the immune microenvironment. Marker genes for 28 types of tumor-infiltrating immune cells were obtained from previous work (33). The raw enrichment scores of each patient were normalized using the formula: [x − min(x)]/[max(x) − min(x)], where x indicated the raw enrichment scores of each cell. The high-risk (n = 148) and low-risk (n = 191) groups from TCGA training cohort were included into the ssGSEA. In addition, 74 samples and 157 samples from the ICGC cohort were analyzed in the same way.

### Statistical Analysis

All statistical analyses were performed using R (version 4.0.3) and its packages. Results associated with two-tailed *P <* 0.05 were considered significant. Where noted, *P*-values were adjusted by the Benjamini–Hochberg correction (21). The “edgeR” and “limma” packages were used to identify DEGs (18–20). Kaplan–Meier analysis was used to generate survival curves for the prognostic analysis, and the log-rank test was used to compare the curves. Independent prognostic factors were identified through univariate and multivariate Cox analyses. ROC curves were constructed using the “pROC” package to evaluate the prognostic value of the risk score (34) in terms of the area under the ROC curve (AUC). Decision curve analysis (DCA) was performed with the source file “stdca.R” (35, 36), obtained from the Memorial Sloan Kettering Cancer Center (https://www.mskcc.org/departments/epidemiology-biostatistics/biostatistics/decision-curve-analysis). The Wilcoxon rank-sum test was used to compare the ssGSEA scores of 28 immune cell types in the low- and high-risk groups. The Pearson test was used to explore correlations among the tumor mutational burden, risk score, and expression of the three immune checkpoints PD-1, PD-L1, and CTLA-4 in terms of log_2_(TPM + 1). Differential expression (in terms of read counts) of the six genes between the two risk groups was analyzed using the “edgeR” package.

## Results

### Demographic and Clinical Characteristics of Patients

**Figure 1:**
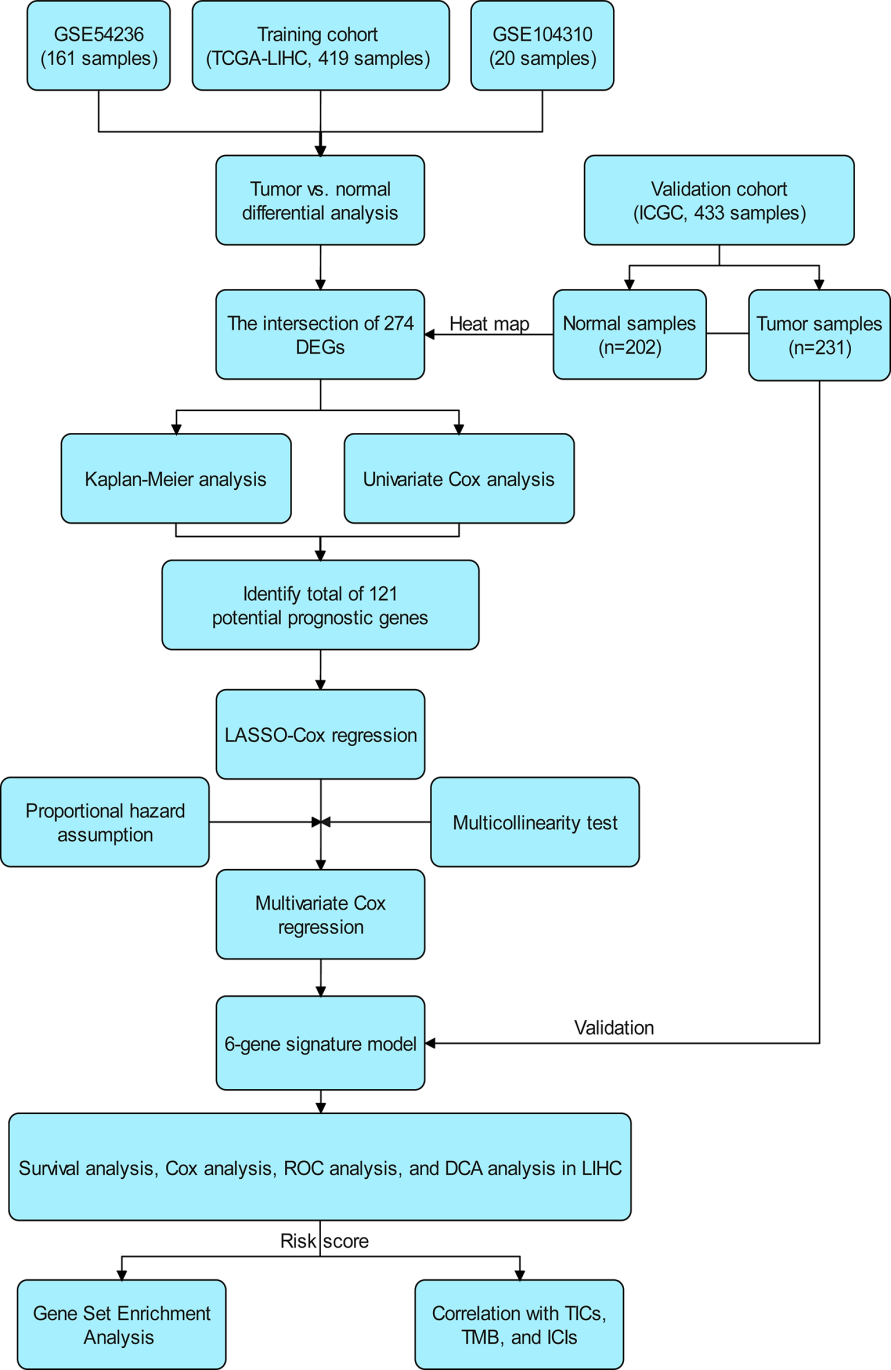
Scheme of the study workflow. LIHC, liver hepatocellular carcinoma; DEGs: differentially expressed genes; LASSO, the least absolute shrinkage and selection operator; ROC, receiver operating characteristic; DCA, decision curve analysis; TMB, tumor mutational burden; TICs, tumor-infiltrating immune cells; ICIs, immune checkpoint inhibitors (PD1, PD-L1, and CTLA4).

The flow chart of the study is shown in **Figure 1**. The 339 cases of HCC in TCGA-LIHC were used as the training cohort. The ICGC (LIRI-JP) data of 231 HCC patients were used as the validation cohort. **Table 1** summarizes the detailed clinical and demographic characteristics of the two cohorts.

**Table 1:** Clinical and demographical characteristics of hepatocellular carcinoma patients included in the study.

Characteristic	Number of patients (%)	
	Training cohort (TCGA-LIHC, n = 339)	Validation cohort (LIRI-JP, n = 231)
**Age (years)**		
<65	208 (61.4)	82 (35.5)
≥65	131 (38.6)	149 (64.5)
**Sex**		
Male	231 (68.1)	170 (73.6)
Female	108 (31.9)	61 (26.4)
**TNM stage**		
I	170 (50.1)	36 (15.6)
II	84 (24.8)	105 (45.5)
III	81 (23.9)	71 (30.7)
IV	4 (1.2)	19 (8.2)
**Histological grade**		
Grade 1	46 (13.6)	NA
Grade 2	166 (49.0)	NA
Grade 3	113 (33.3)	NA
Grade 4	12 (3.5)	NA
Unknown	2 (0.6)	NA
**Ishak score**		
0–4	124 (36.6)	NA
5–6	74 (21.8)	NA
Unknown	141 (41.6)	NA
**Child–Pugh grade**		
A	207 (61.1)	NA
B–C	21 (6.2)	NA
Unknown	111 (32.7)	NA
**Alpha fetoprotein (ng/ml)**		
≤200	191 (56.3)	NA
>200	72 (21.2)	NA
Unknown	76 (22.4)	NA

Values are n (%), unless otherwise noted.

NA, not applicable; TNM, tumor node metastasis stage.

### DEG Analysis

**Figure 2:**
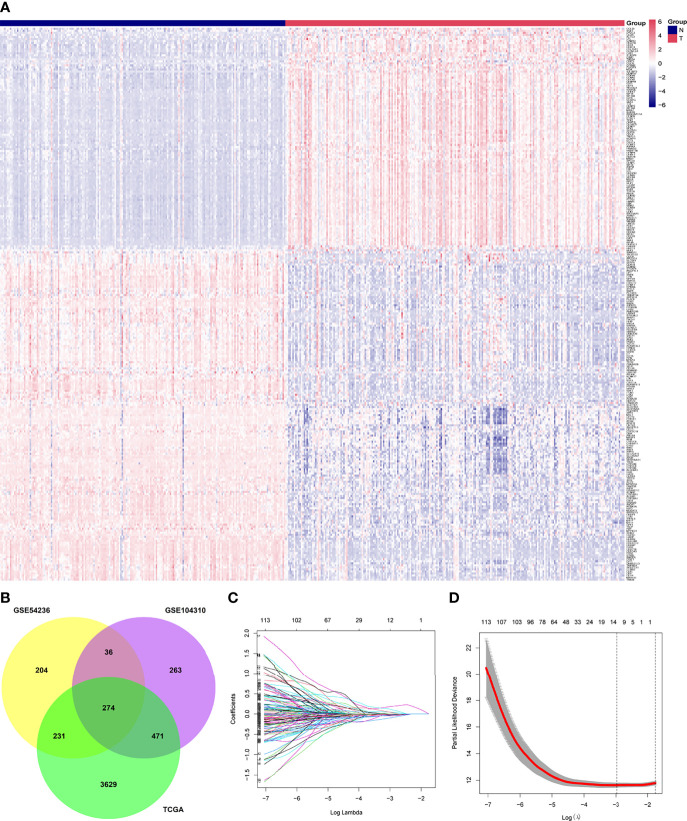
Identification of differentially expressed genes between tumor (T) and adjacent normal (N) tissues and potential prognostic genes in the The Cancer Genome Atlas, GSE54236, and GSE104310 datasets. **(A)** Overlapping differentially expressed genes between tumor and adjacent normal tissues, presented as a clustering heatmap in the ICGC cohort. **(B)** Venn diagram showing differentially expressed genes between tumor and adjacent normal tissue in the three datasets included in the study. **(C)** LASSO coefficient profiles of 121 genes related to prognosis of hepatocellular carcinoma patients. **(D)** Ten-fold cross-validation for selecting the optimal lambda (λ) in the LASSO algorithm. LASSO, the least absolute shrinkage and selection operator.

In our gene expression analysis, we found 4,605 DEGs (3,507 upregulated and 1,098 downregulated) between primary tumor and normal control tissues in TCGA dataset, 1,044 (427 upregulated and 617 downregulated) in GSE104310, and 745 (267 upregulated and 478 downregulated) in GSE54236. We identified 274 DEGs common to the three datasets, which we selected for further analysis. We removed the genes PVALB and GDF2 from this set because of missing values in the ICGC cohort. The final 272 overlapping genes are shown in a heat map in **Figure 2A**. The Venn plot showing DEGs and overlapping genes from the three datasets is presented in **Figure 2B**.

### Construction of a Prognostic Signature From the Training Cohort

A total of 121 genes were identified by Kaplan–Meier and univariate Cox analyses as potential prognostic genes (**Supplementary Table 1**). We analyzed these genes by LASSO Cox regression and calculated the regression coefficients (**Figure 2C**). When 12 genes were included, the Lasso–Cox regression function performed better (**Figure 2D**), with λ = 0.05103363. These 12 genes and the regression coefficients are shown in **Supplementary Table 2**. The 12 genes were included in the multivariate Cox regression, and both-direction stepwise regression was implemented to further select genes according to the lowest Akaike Information Criterion value (37). Subsequently, we checked for violations of the proportional hazard assumptions (**Supplementary Table 3**), which led to exclusion of the gene CENPA because its *P <* 0.05 (**Supplementary Figure 1**). In addition, we found that the multicollinearity assumption was not violated, with all VIF < 2 (**Supplementary Table 4**). Finally, a six-gene expression signature was established by multivariate Cox regression (**Figure 6D**). These six genes and their corresponding regression coefficients are shown in **Supplementary Table 5**.

### Prognostic Value of the Six-Gene Expression Signature in the Training and Validation Cohorts

The following formula including the six selected genes was used to calculate the risk score of each patient: risk score = PZP ∗ (−0.270388554) + HMMR ∗ (0.451615489) + LCAT ∗ (−0.134842021) + GRAMD1C ∗ (−0.262454435) + LPL ∗ (0.216649959) + ANGPTL1 ∗ (−0.152772802). Based on the gene expression level [log_2_(TPM + 1)] and the risk coefficient of each gene, the *predict* function of the “stats” package in R was used to obtain the risk score of each patient (25). According to the optimal cut-off risk score (1.195033), patients in TCGA cohort were assigned to a high-risk group (n = 148) or a low-risk group (n = 191) based on overall survival. Analogously, patients in TCGA cohort were divided according to the optimal cut-off risk score (0.8979665) into a high-risk group (n = 192) or a low-risk group (n = 147) based on progression-free interval. The ICGC validation cohort was also divided according to the optimal TCGA cohort cut-off value into a high-risk group (n = 74) or low-risk group (n = 157) based on overall survival.

**Figure 3:**
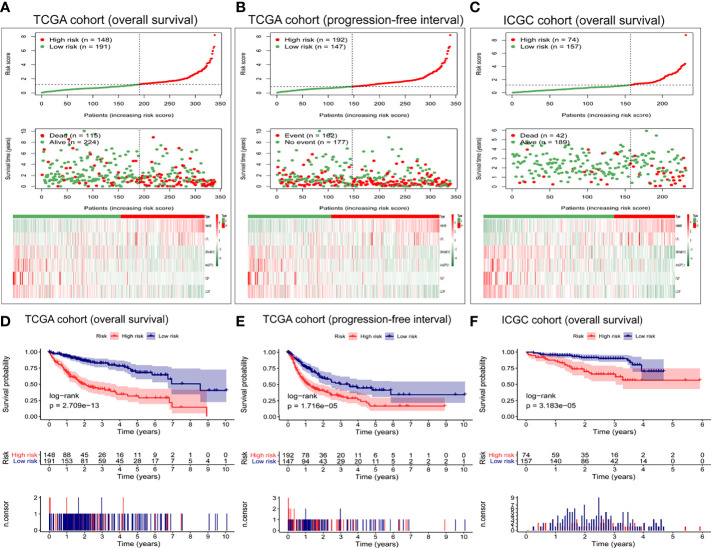
Evaluation of the prognostic capacity of the six-gene expression signature. Distribution of six-gene risk scores, survival time, survival state, and the six-gene expression heat map between the low- and high-risk groups based on **(A)** overall survival or **(B)** progression-free interval in TCGA cohort, or on **(C)** overall survival in the ICGC cohort. Kaplan-Meier survival analysis based on **(D)** overall survival and **(E)** progression-free interval in TCGA cohort. Kaplan-Meier survival analysis based on **(F)** overall survival in the ICGC cohort. Differences between survival curves were assessed for significance using the log-rank test.

**Figure 3A** shows the distribution of risk scores and overall survival of patients in TCGA cohort, as well as the heat map of the expression levels of the six-gene expression signature. As the risk score increased, the number of deaths among high-risk patients increased, and survival became shorter. The heat maps show that expression of GRAMD1C, ANGPTL1, PZP, and LCAT was low in high-risk patients, while expression of HMMR and LPL was higher in high-risk patients than in low-risk ones.

Moreover, we explored the performance of the six-gene expression signature in predicting the progression-free interval in TCGA cohort. As shown in **Figure 3B**, the high-risk group presented more death events and shorter survival. Next, we verified the predictive power of these six gene markers on the overall survival in the ICGC cohort. The high-risk group had more deaths and shorter survival than low-risk patients (**Figure 3C**). The expression pattern of the six-gene expression signature in the ICGC cohort was consistent with that in TCGA cohort.

As shown in **Figure 3**, the survival curve of TCGA cohort was different between high- and low-risk groups (*P* = 2.709e−13, **Figure 3D**). Similarly, the Kaplan–Meier survival curve showed that the progression-free interval was shorter in the high-risk group than in the low-risk group (*P* = 1.716e−05, **Figure 3E**). The robustness of the six-gene expression signature in HCC patients was tested in the ICGC validation cohort. Patients in the high-risk group presented shorter overall survival than patients in the low-risk group (*P* = 3.183e−05) (**Figure 3F**), consistent with the results from TCGA dataset.

**Figure 4:**
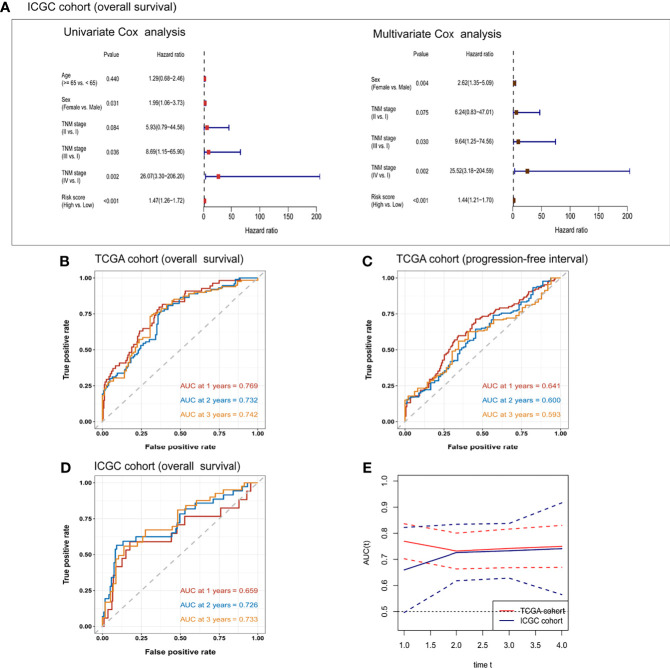
Univariate and multivariate Cox regression analyses of the risk score and clinical/demographic indicators related to **(A)** overall survival in the ICGC cohort. Receiver operating characteristic curves for the prediction of one-, two-, and three-year **(B)** overall survival or **(C)** progression-free interval in TCGA cohort, or **(D)** overall survival in the ICGC cohort. **(E)** Time-dependent area under the receiver operating curve (AUC) and the corresponding 95% confidence intervals for predicting overall survival at one, two, and three years. The red line corresponds to TCGA cohort and the blue line corresponds to the ICGC cohort.

In TCGA cohort, the risk score was an independent prognostic factor for overall survival based on univariate analysis [hazard ratio (HR) 1.61, 95% confidence interval (CI) 1.45–1.79, *P <* 0.001] and multivariate analysis (HR 1.56, 95% CI 1.40–1.74, *P <* 0.001; **Table 2**). Risk score was also a predictor of progression-free interval in the univariate analysis (HR 1.34, 95% CI 1.20–1.51, *P <* 0.001) and multivariate analysis (HR 1.25, 95% CI 1.10–1.41, *P <* 0.001; **Table 3**). In the ICGC cohort, the risk score was validated as an independent predictor of overall survival in the univariate analysis (HR 1.47, 95% CI 1.26–1.72, *P <* 0.001) and multivariate analysis (HR 1.44, 95% CI 1.21–1.70, *P <* 0.001; **Figure 4A**). These results suggest that the risk score is an independent predictor of overall survival and progression-free interval. Moreover, tumor stage may also be an independent prognostic factor.

**Table 2:** Cox proportional hazards regression model of overall survival in the cancer genome atlas cohort.

Variable	Univariate analysis		Multivariate analysis	
	HR (95% CI)	P*	HR (95% CI)	P*
Age (≥65 *vs*. <65 years)	1.23 (0.85, 1.78)	0.273	–	–
Sex (female *vs*. male)	1.26 (0.87, 1.84)	0.228	–	–
TNM stage (II *vs*. I)	1.42 (0.87, 2.32)	0.16	1.07 (0.65, 1.76)	0.792
TNM stage (III *vs*. I)	2.72 (1.78, 4.15)	**<0.001**	2.06 (1.33, 3.21)	**0.001**
TNM stage (IV *vs*. I)	5.44 (1.68, 17.6)	**0.005**	5.35 (1.65, 17.4)	**0.005**
Histological grade (3–4 *vs*. 1–2)	1.14 (0.78, 1.67)	0.489	–	–
Ishak score (5–6 *vs*. 0–4)	0.87 (0.5, 1.5)	0.612	–	–
Child–Pugh grade (B–C *vs*. A)	1.66 (0.82, 3.36)	0.159	–	–
Alpha fetoprotein (>200 *vs*. ≤200 ng/ml)	1.01 (0.61, 1.66)	0.979	–	–
Risk score (high *vs*. low)	1.61 (1.45, 1.79)	**<0.001**	1.56 (1.4, 1.74)	**<0.001**

95% CI, 95% confidence interval; HR, hazard ratio; TNM, tumor node metastasis stage.

*Statistically significant p values are given in bold, P < 0.05.

**Table 3:** Cox proportional hazards regression model of progression-free interval in the cancer genome atlas cohort.

Variable	Univariate analysis		Multivariate analysis	
	HR (95% CI)	P*	HR (95% CI)	P*
Age (≥65 *vs*. <65 years)	0.91 (0.66, 1.26)	0.572	–	–
Sex (female *vs*. male)	1.04 (0.75, 1.44)	0.828	–	–
TNM stage (II *vs*. I)	1.92 (1.31, 2.82)	**0.001**	1.63 (1.10, 2.43)	**0.015**
TNM stage (III *vs*. I)	2.77 (1.91, 4.01)	**<0.001**	2.45 (1.67, 3.59)	**<0.001**
TNM stage (IV *vs*. I)	4.04 (0.98, 16.7)	0.054	4.06 (0.98, 16.8)	0.053
Histologic grade (3–4 *vs*. 1–2)	1.14 (0.83, 1.57)	0.41	–	–
Ishak score (5–6 *vs*. 0–4)	1.18 (0.80, 1.75)	0.405	–	–
Child–Pugh grade (B–C *vs*. A)	1.31 (0.70, 2.45)	0.399	–	–
Alpha fetoprotein (>200 *vs*. ≤200 ng/ml)	1.05 (0.70, 1.57)	0.81	–	–
Risk score (high *vs*. low)	1.34 (1.20, 1.51)	**<0.001**	1.25 (1.10, 1.41)	**<0.001**

95% CI, 95% confidence interval; HR, hazard ratio; TNM stage, tumor node metastasis stage.

*Statistically significant p values are given in bold, P < 0.05.

To further confirm the prognostic potential of the six-gene expression signature in HCC patients, we conducted time-dependent ROC analyses in the training and validation cohorts. As shown in **Figure 4B**, the AUC of the multivariate regression model in TCGA cohort was 0.769 for one-year overall survival, 0.732 for two-year survival, and 0.742 for three-year survival. The AUCs of the six-gene prognostic signature for predicting progression-free interval in the training cohort were 0.641 for one year, 0.600 for two years, and 0.593 for three years (**Figure 4C**). The corresponding AUCs in the prediction of overall survival in the ICGC validation cohort were 0.659, 0.726, and 0.733 (**Figure 4D**). The time-dependent AUCs and their 95% confidence intervals are shown in **Figure 4E**.

**Figure 5:**
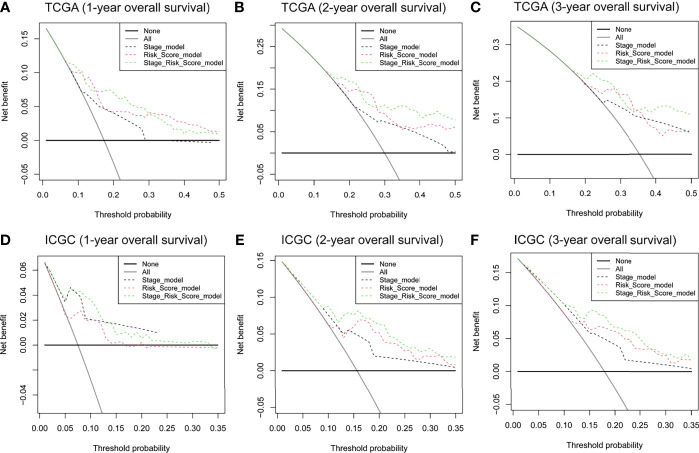
DCA curves to assess the ability of TNM stage, risk score, and their combination to predict one-, two-, and three-year overall survival in **(A–C)** TCGA and **(D–F)** ICGC cohorts. DCA, decision curve analysis. TNM stage, tumor node metastasis stage.

DCA was applied to compare the clinical utility of the risk model to that of TNM staging for predicting one-, two-, and three-year overall survival of HCC patients in TCGA (**Figures 5A–C**) and ICGC cohorts (**Figures 5D–F**). The prognostic risk model proved better than traditional TNM staging in all cases. Even better performance was obtained by combining the TNM stage with our risk score model.

**Figure 6:**
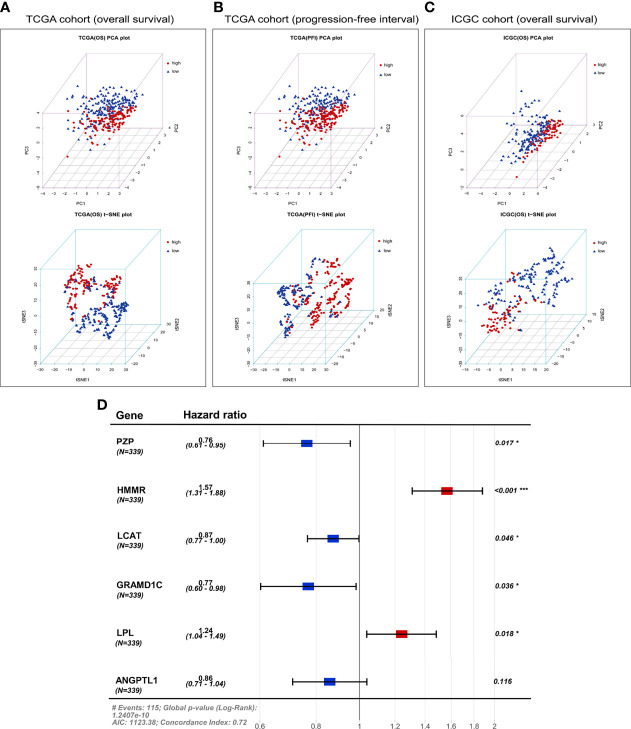
Principal component analysis (PCA) and t-distributed stochastic neighbor embedding (t-SNE) analysis based on the six-gene expression signature. PCA and t-SNE plot of **(A)** overall survival or **(B)** progression-free interval in TCGA cohort, or **(C)** overall survival in the ICGC cohort. **(D)** Forest plot of the six genes in the prognostic risk model and overall survival in TCGA cohort. AIC, Akaike Information Criterion.

PCA and t-SNE were used to assess the classification ability of the model and to visualize data distribution in discrete directions by reducing the data from high to low dimensionality. In TCGA cohort, we used PCA and t-SNE to test the distribution of patients into two subgroups of low-dimensional data. **Figure 6** presents the results of the PCA and t-SNE using the expression of the six signature genes in the prognostic risk model. **Figure 6A** shows that patients in different risk groups were clearly distinguished. A similar analysis in three directions was conducted for progression-free interval in TCGA cohort (**Figure 6B**) and the ICGC cohort (**Figure 6C**).

### Correlations Among Risk Score, Tumor Mutational Burden, Immune Checkpoint Genes, and Six-Gene Expression Signature in High- and Low-Risk Groups

**Figure 7:**
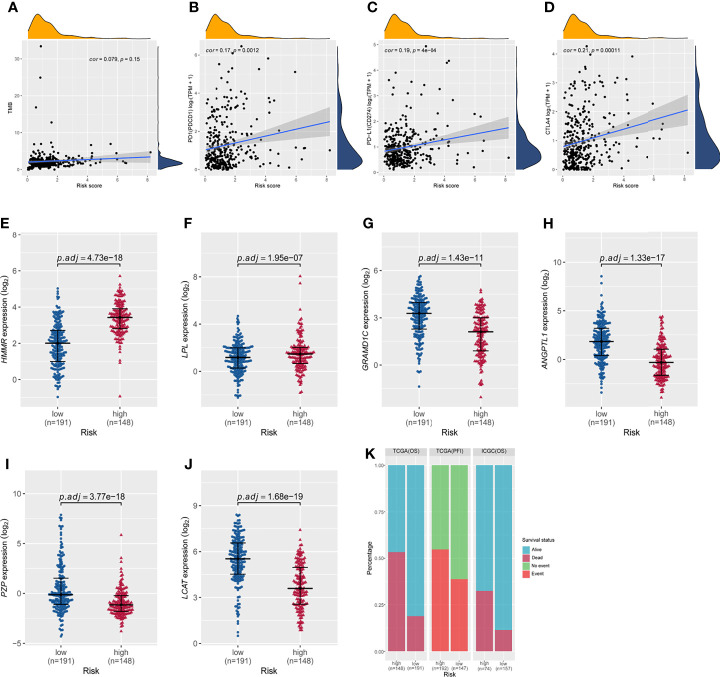
Correlations of the risk score based on the six-gene prognostic model with tumor mutational burden; expression of PD1, PD-L1, and CTLA4; and the differential expression of the six signature genes between the high- and low-risk groups in TCGA cohort based on overall survival. **(A)** The risk score did not correlate with tumor mutational burden. The risk score did not correlate with **(B)** PD-1 expression, **(C)** PD-L1 expression, or **(D)** CTLA-4 expression. **(E–J)** Expression of HMMR, LPL, GRAMD1C, ANGPTL1, PZP, and LCAT in the high- and low-risk groups of TCGA cohort based on overall survival. *P* values were adjusted using the Benjamini-Hochberg method. **(K)** Distribution of patient survival status between the high- and low-risk groups.

Correlation analyses were performed to assess relationships among risk score, tumor mutational burden, and expression of the three immune checkpoint genes PD-1, PD-L1, and CTLA-4. In TCGA cohort, the correlation between the risk score and tumor mutational burden was analyzed using the Pearson test. The results showed that the six-gene expression signature did not correlate with tumor mutational burden (Pearson *cor* = 0.079, *P* = 0.15, **Figure 7A**). Correlations between expression of PD-1, PD-L1, or CTLA-4 and the risk score of the six-gene expression signature are shown in **Figure 7**. Based on the overall survival in TCGA cohort, the risk score showed no correlation with PD-1 (Pearson *cor* = 0.17, *P* = 0.0012, **Figure 7B**), PD-L1 (Pearson *cor* = 0.19, *P* = 4e−04, **Figure 7C**), or CTLA-4 (Pearson *cor* = 0.21, *P* = 0.00011, **Figure 7D**). In addition, the expression of HMMR and LPL was markedly higher in the high-risk group than in the low-risk group (**Figures 7E, F**), while the expression of GRAMD1C, ANGPTL1, PZP, and LCAT was significantly down-regulated in high-risk patients (**Figures 7G–J**). Survival was significantly different between the high- and low-risk groups in both TCGA and ICGC cohorts (**Figure 7K**). These results indicate that the prognostic risk model can effectively stratify HCC patients into high- and low-risk groups, consistent with previous results.

### Gene Set Enrichment Analysis With the Six-Gene Signature

**Figure 8:**
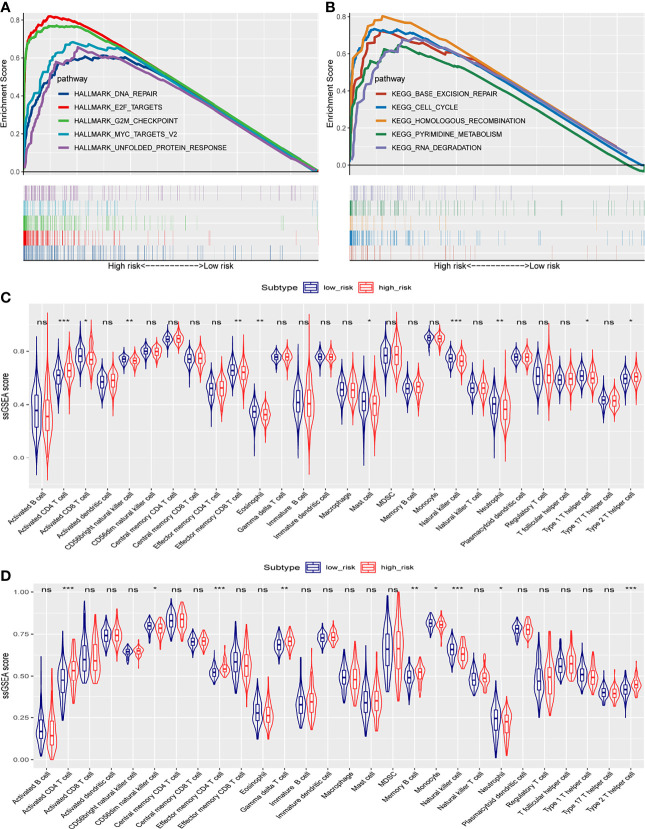
Gene set enrichment analysis in TCGA cohort, and ssGSEA scores between the high- and low-risk groups in TCGA and ICGC cohorts. **(A)** Significantly enriched pathways in the Hallmark gene sets between the two risk groups in TCGA cohort. **(B)** Enriched pathways in the KEGG analysis between the two risk groups in TCGA cohort. The enrichment scores of 28 types of tumor-infiltrating immune cells are shown in the violin plot for **(C)** TCGA cohort and **(D)** the ICGC cohort. ns, not significant; *, *P* < 0.05; **, *P* < 0.01; ***, *P* < 0.001.

To understand the biological functions and pathways involved in HCC patients, we performed GSEA between the high- and low-risk groups. In the high-risk group, significantly enriched gene sets of the Hallmark Collection were found in the following signaling pathways: unfolded protein response, G2M checkpoint, E2F targets, MYC targets V2, and DNA repair (**Figure 8A** and **Supplementary Table 6**). For the KEGG gene sets defined by the Molecular Signatures Database, enriched pathways in the high-risk group were mostly related to base excision repair, cell cycle, homologous recombination, pyrimidine metabolism, and RNA degradation (**Figure 8B** and **Supplementary Table 7**). These results suggest that signature genes overexpressed in HCC are closely associated with cancer-related pathways.

### Correlation Between the Risk Score and Immune Status

To investigate the correlation of the risk score with the immune microenvironment of HCC tumors, we quantified the enrichment of 28 types of immune cells. High- and low-risk groups in TCGA cohort (based on overall survival) showed differences in enrichment of activated CD4^+^ T cells, activated CD8^+^ T cells, CD56-bright natural killer cells, effector memory CD8^+^ T cells, eosinophils, mast cells, natural killer cells, neutrophils, type 1 T helper cells, and type 2 T helper cells (**Figure 8C**). Scores were lower in the high-risk group for the following cell types: activated CD8^+^ T cells, CD56-bright natural killer cells, effector memory CD8^+^ T cells, eosinophils, mast cells, natural killer cells, neutrophils, and type 1 T helper cells. Scores were higher in the high-risk group for the following cell types: activated CD4^+^ T cells and type 2 T helper cells.

In the ICGC validation cohort, the two risk groups differed in scores for activated CD4^+^ T cells, CD56-dim natural killer cells, effector memory CD4^+^ T cells, gamma delta T cells, memory B cells, monocytes, natural killer cells, neutrophils, and type 2 T helper cells (**Figure 8D**). Consistent with the results from TCGA dataset, the high-risk ICGC group showed higher scores for activated CD4^+^ T cells and type 2 T helper cells than the low-risk group.

## Discussion

In this work, we identified and validated a novel six-gene expression prognostic signature for HCC patients based on bioinformatic analyses of publicly available data. The prognostic model proposed is based on six differentially expressed genes (PZP, HMMR, LCAT, GRAMD1C, LPL, and ANGPTL1). In our prognostic model, overexpression of PZP, LCAT, GRAMD1C, and ANGPTL1 was associated with better prognosis in HCC patients, while overexpression of HMMR and LPL was associated with worse prognosis. The prognostic performance of the six-gene expression signature in the present study was robust in both TCGA cohort and the ICGC validation cohort. The AUC for one-, two-, and three-year overall survival was high, but the model proved poor at predicting progression-free interval. For both overall survival and progression-free interval, the risk score was an independent prognostic factor in HCC patients, and the prognosis of patients in the high-risk group was significantly worse than that in the low-risk group. Based on these results, our risk score may be useful as a predictor of HCC patient survival.

The genes in our signature have previously been linked to HCC or other cancers. Human pregnancy zone protein (PZP) is abundant in the serum of women in late pregnancy, and its levels are closely related to immunosuppression during pregnancy (38). Downregulation of PZP in HCC tissues correlates with poor prognosis (39), which is consistent with our results; PZP overexpression inhibits proliferation, invasion, and migration of HCC cells, and the downregulation of PZP in HCC has been linked to hypermethylation of the gene (40). These observations suggest that overexpression of PZP can play a protective role in patients with HCC. Future studies should clarify the role of PZP in HCC.

HMMR, also known as RHAMM, is associated with neoplastic processes in multiple tumor types, and it is a breast cancer susceptibility gene (41). HMMR induces the epithelial-mesenchymal transition, promoting resistance to chemotherapy (42). HMMR is overexpressed in lung adenocarcinoma tissues, and HMMR knockdown in lung adenocarcinoma inhibits cell proliferation, migration and invasion, while increasing apoptosis (43). Future work should explore the role of HMMR in HCC.

Lecithin-cholesterol acyltransferase (LCAT) is produced by the liver and secreted into the circulation, and patients with liver disease show reduced LCAT activity (44). Lower LCAT expression has been linked to poor HCC prognosis (45), in agreement with our data.

Similarly, low expression of GRAMD1C correlates with poor prognosis, worse tumor pathology and distant metastasis in patients with kidney renal clear cell carcinoma (46). Our results highlight the need to explore the role of GRAMD1C in HCC.

Lipoprotein lipase (LPL) plays a central role in the hydrolysis of circulating triglycerides present in chylomicrons and very-low-density lipoproteins (47). Overexpression of LPL has been linked to poor prognosis in HCC patients, and silencing the gene inhibits proliferation of HCC cell lines (48). The ability of LPL overexpression to worsen prognosis may relate to the protein’s ability to enhance uptake of exogenous lipids, which activates HCC cell proliferation (49).

ANGPTL1, a member of the angiopoietin-like protein family, inhibits tumor angiogenesis and metastasis (50, 51). In HCC cells, ANGPTL1 promotes apoptosis by inhibiting the STAT3/Bcl-2-mediated anti-apoptotic pathway, and it downregulates the transcription factors SNAIL and SLUG, thereby decreasing cell migration and invasion (52). ANGPTL1 overexpression inhibits the MET receptor-AKT/ERK-Egr-1-Slug signaling cascade, repressing the epithelial–mesenchymal transition and thereby counteracting sorafenib resistance and cancer stemness in HCC cells (53). Consistently with these observations, we found that ANGPTL1 expression was lower in high-risk patients than in low-risk patients.

However, our study presents several limitations. First, our results are based on bioinformatic analyses of publicly available data, and clinical validation is needed. In particular, future experiments should elucidate the molecular mechanisms of the six signature genes in HCC. Second, some genes associated with prognosis might have been excluded from the study and from our prognostic risk model, perhaps as a result of the rigorous thresholds that we applied during screening. Third, in some cases we used the KNN algorithm to fill in missing values for gene expression, which might have introduced bias.

## Conclusion

Despite these limitations, the present work provides a comprehensive analysis of gene expression data in HCC from TCGA, GEO, and ICGC databases. We established a robust six-gene prognostic model for HCC patients. The signature genes may serve as biomarkers for HCC, providing patients with personalized prognostic prediction.

## Data Availability Statement

Publicly available datasets were used in this study. These data can be found here: The Cancer Genome Atlas (TCGA-LIHC, https://gdc.cancer.gov/), the corresponding clinical and survival data of TCGA cohort (TCGA Liver Cancer (LIHC) (19 datasets), https://xenabrowser.net/), Gene Expression Omnibus (GSE54236 and GSE104310, https://www.ncbi.nlm.nih.gov/geo/), and International Cancer Genome Consortium (LIRI-JP, https://icgc.org/).

## Author Contributions

ZCY conceived and designed the study, performed the bioinformatic analysis, conducted the statistical analysis, analyzed the data, and wrote the manuscript. MLH, LFH, and LXW revised the manuscript. YMZ revised the manuscript and provided financial support. All authors contributed to the article and approved the submitted version.

## Funding

This research did not receive any specific grant from funding agencies in the public, commercial, or not-for-profit sectors.

## Conflict of Interest

The authors declare that the research was conducted in the absence of any commercial or financial relationships that could be construed as a potential conflict of interest.

## Publisher’s Note

All claims expressed in this article are solely those of the authors and do not necessarily represent those of their affiliated organizations, or those of the publisher, the editors and the reviewers. Any product that may be evaluated in this article, or claim that may be made by its manufacturer, is not guaranteed or endorsed by the publisher.

## Abbreviations

AUC, area under the receiver operating characteristic curve; DCA, decision curve analysis; DEGs, differentially expressed genes; GEO, Gene Expression Omnibus; GSEA, gene set enrichment analysis; HCC, hepatocellular carcinoma; ICGC, International Cancer Genome Consortium; LASSO, least absolute shrinkage and selection operator; ROC, receiver operating characteristic; ssGSEA, single-sample gene set enrichment analysis; TCGA, The Cancer Genome Atlas; TPM, transcripts per million.
