# Supplementary figures and images for: Identification and Validation of a Novel Six-Gene Expression Signature for Predicting Hepatocellular Carcinoma Prognosis
Source: Front Immunol. 2021 Dec 1;12:723271. doi: 10.3389/fimmu.2021.723271 (PMC8671815; doi:10.3389/fimmu.2021.723271)

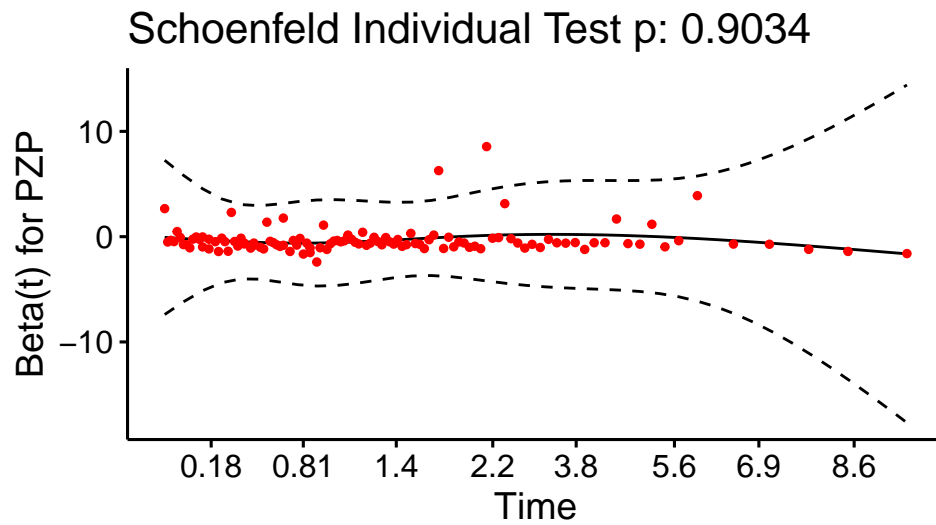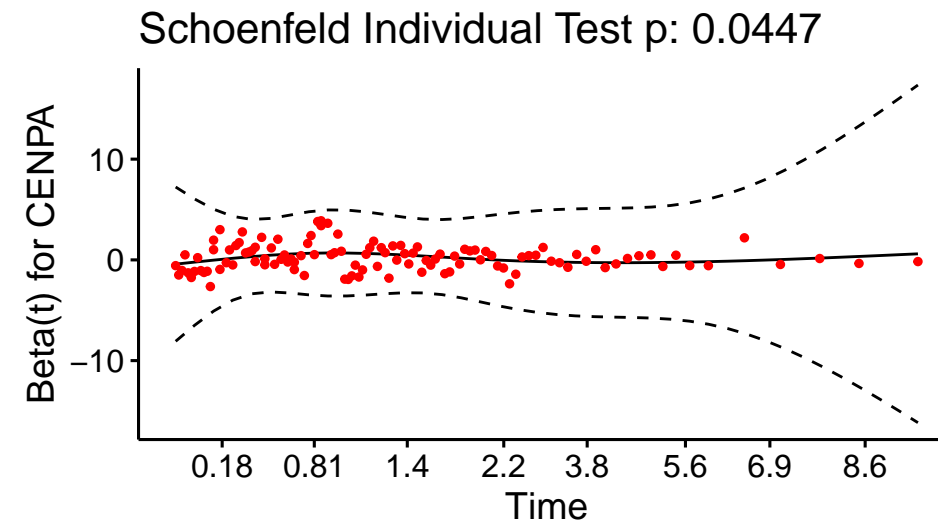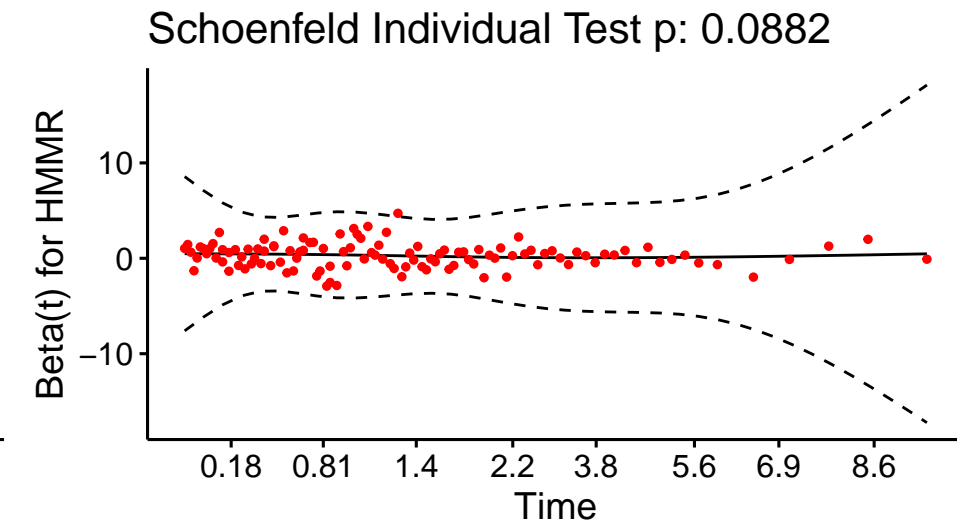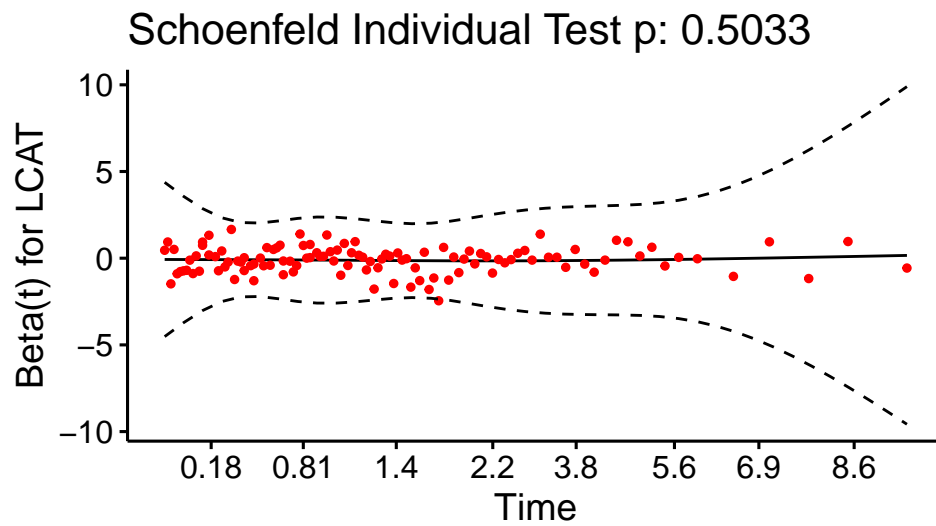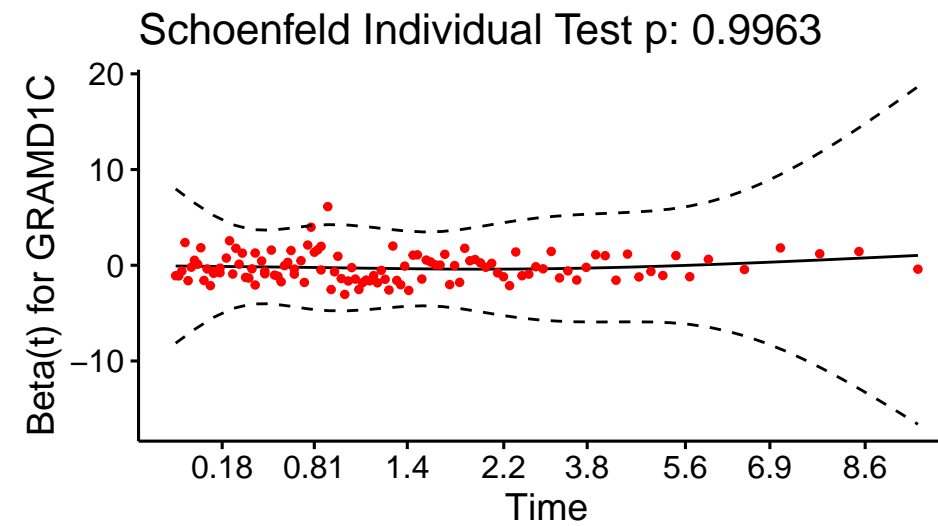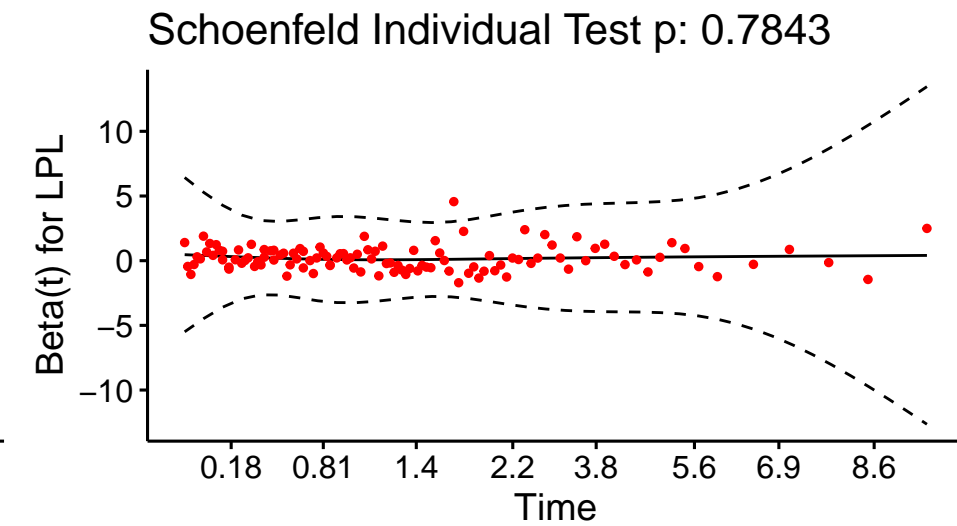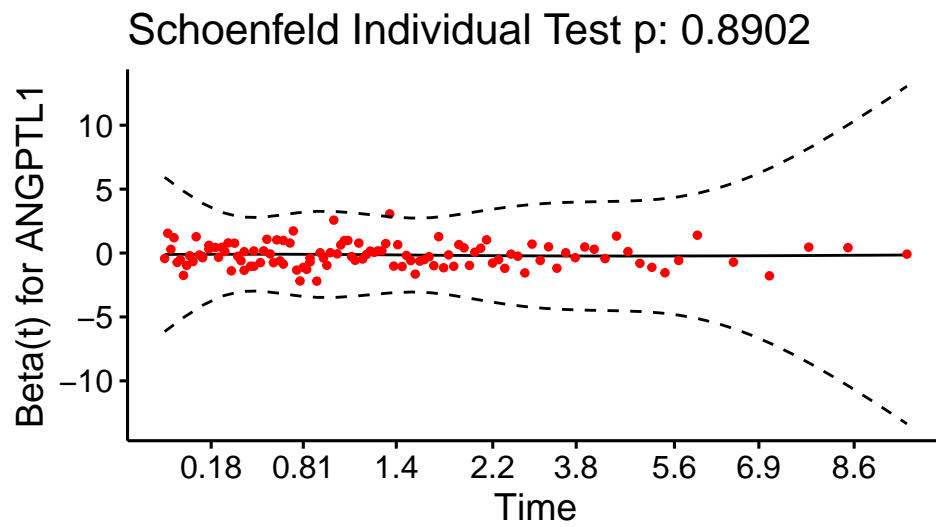

Supplement: Supplementary file 1 [file DataSheet_1.zip › Supplementary Figure 1.pdf]
